# Biomarkers for diagnosis and prognosis of myelin oligodendrocyte glycoprotein antibody-associated disease - review article

**DOI:** 10.3389/fimmu.2025.1594960

**Published:** 2025-06-06

**Authors:** Tamang Sapana, Zhihong Zhuo

**Affiliations:** ^1^ Department of Pediatrics, The First Affiliated Hospital of Zhengzhou University, Zhengzhou, Henan, China; ^2^ Henan Provincial Key Laboratory of Childhood Epilepsy and Immunology, Zhengzhou, Henan, China

**Keywords:** myelin oligodendrocyte glycoprotein antibody-associated disease (MOGAD), CNS demyelinating disorders, biomarkers, multiple sclerosis, neuromyelitis optica spectrum disorders, multiple sclerosis (MS), neuromyelitis optica spectrum disorders (NMOSD)

## Abstract

Myelin oligodendrocyte glycoprotein antibody-associated disease (MOGAD) is a complex inflammatory central nervous system disorder affecting mainly children and young adults. Its diverse clinical spectrum often resembles other neurological diseases like multiple sclerosis (MS) or neuromyelitis optica spectrum disorder (NMOSD). Accurate diagnosis and monitoring are challenging due to its heterogeneous presentation and lack of specific biomarkers. In addition, disease-modifying therapies are well-established for treating MS and NMOSD. In contrast, the absence of approved therapies for MOGAD remains a significant challenge in clinical management and should become a priority in future research efforts. This review explores existing literature on biomarkers in MOGAD, including clinical, radiological, and laboratory parameters along with novel biomarkers and the future challenges that can facilitate personalized therapeutic approaches in MOGAD. A comprehensive understanding of these biomarker profiles is crucial for optimizing patient care and advancing therapeutic strategies in neuroinflammatory disorders.

## 1 Introduction

Myelin oligodendrocyte glycoprotein antibody-associated disorder (MOGAD) was a newly identified disorder of neuroimmunology condition with evolving diagnostic and management guidelines. Initially, classified under seronegative neuromyelitis optica spectrum disorders (NMOSD) (1), MOG antibodies were later recognized as distinct markers in cases negative for AQP4-IgG. MOGAD has a global prevalence of 1.3–2.5 per 100,000 and an annual incidence of 1.6–3.4 per million (2). According to recent diagnostic criteria of 2023 internaltional consensus (3), MOGAD clinically presents with diverse manifestations including, optic neuritis (ON), transverse myelitis, acute disseminated encephalomyelitis (ADEM), and cortical encephalitis along with positive MOG-IgG seropositivity via cell-based assay (CBA). ON is a common initial symptom in MOGAD especially in adults, while children often present with ADEM. Symptoms include vision loss, retro-orbital pain, and color vision loss. Bilateral ON is more frequent in MOGAD patients, and relapses are typically unilateral (3). Currently, there are limited clinical, and radiological biomarkers exist to predict disease course, severity, or relapses. Identifying accessible and reproducible biomarkers is essential for guiding diagnosis and treatment. Our study compiles existing research on biomarkers relevant to MOGAD to improve clinical diagnosis, management strategies, and relapse prevention while highlighting future research challenges (**Table 1**).

**Table 1:** The summary table outlines key biomarkers categorized by diagnostic, prognostic, monitoring, and treatment response domains.

Category	Biomarker	Sensitivity/Specificity	Clinical Utility	Limitations
**Diagnosis**	**Serological**: MOG-IgG (Cell-based Assay)	High/High (Live assay preferred)	Confirmatory test for MOGAD	False positives in MS; seronegativity in some
	**CSF**: Pleocytosis, OCBs	Moderate/Low	Supportive when serum negative	Non-specific; overlap with other neuroimmunological conditions
	**Radiology**: MRI (optic nerve swelling, LETM)	Variable	Early diagnosis, phenotype correlation	Not pathognomonic
**Disease Monitoring & Relapse Prediction**	MOG-IgG Titer Trend	N/A	Relapse risk assessment	Persistent seropositivity, not always predictive
	Relapse Patterns (ON, TM, ADEM)	N/A	Guides follow-up frequency	Variable individual trajectories
	MRI (Silent or new lesions)	Moderate	Monitoring progression	May not correlate with symptoms
	Novel biomarkers: PLR, NLR, sTREM2, EGFR/NFLs	Under evaluation	Acute phase, relapse correlation	Requires validation in future
**Treatment Response**	Steroid Tapering Schedule	N/A	Predict relapse risk	Individual variability
	IVIG, RTX, Tocilizumab Response	N/A	Tailors long-term therapy	Not all respond; no definitive predictor
	Imaging (OCT, mVEP)	Moderate	Functional outcome tracking	Accessibility, cost
**Prognosis/Severity**	sNfL, sGFAP	Moderate	Disease activity, relapse prediction	Limited pediatric data
	mRNA Markers (EGFR, NFL)	Experimental	Severity in ADEM phenotype	Undergoing research
	Imaging Severity (FLAMES, H-sign, atrophy)	Moderate	Acute attack evaluation	Not unique to MOGAD
	Immunophenotyping (Treg, Th17)	Experimental	Pathogenic mechanism insights	Not routinely available

## 2 Biomarkers

### 2.1 Clinical biomarkers

Myelin oligodendrocyte glycoprotein antibody-associated disorder (MOGAD) is a distinct inflammatory demyelinating disorder of the central nervous system (CNS), separate from multiple sclerosis (MS) and neuromyelitis optica spectrum disorders (NMOSD). The diagnostic criteria of MOGAD including optic neuritis, transverse myelitis, ADEM, cerebral deficits, brainstem syndromes, and cerebral cortical encephalitis as cardinal features (3).

**Figure 1:**
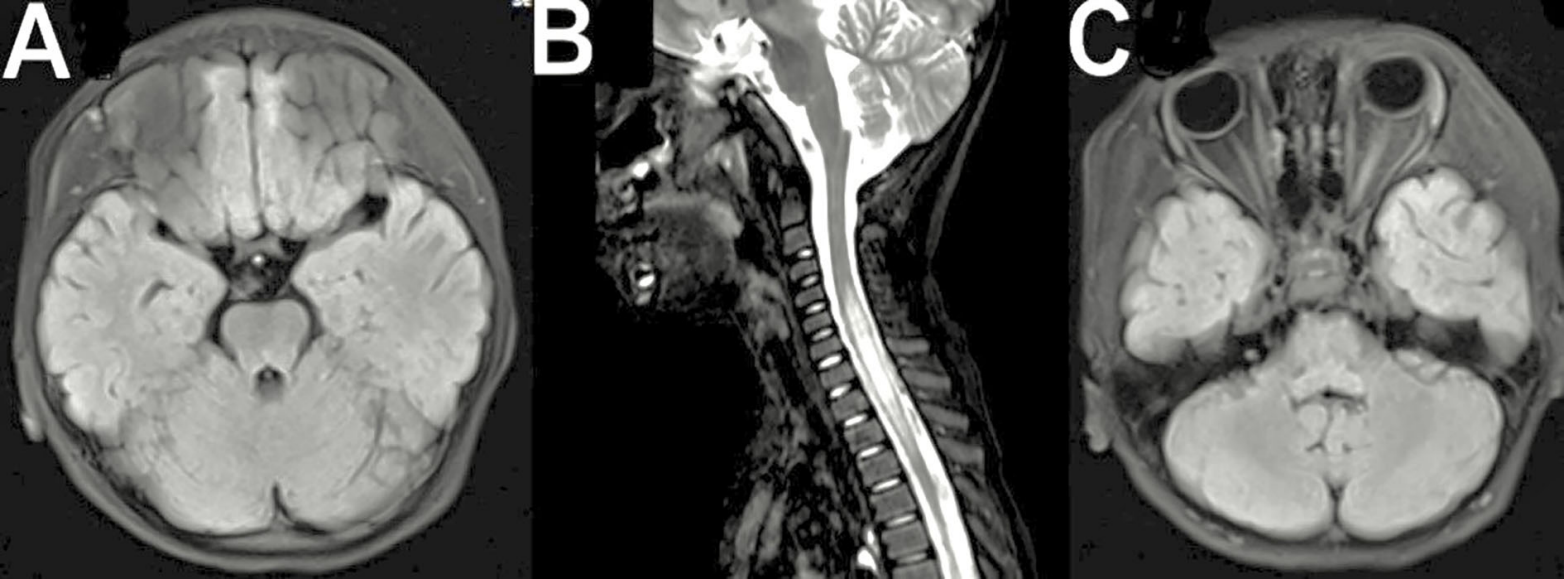
**(A)** Axial FLAIR brain MRI showing hyperintense lesions in the brainstem and cerebellum. **(B)** Sagittal T2-weighted MRI of the cervical spine demonstrating longitudinally extensive transverse myelitis (LETM). **(C)** Axial FLAIR MRI indicating possible optic nerve involvement.

Optic neuritis is the most common initial symptom, particularly in adults. In contrast, children, especially those under the age of 12, often present with ADEM, with or without optic nerve involvement (2, 4). Loss of central acuity, retro-orbital pain (which some patients perceive as a headache) (5), loss of color vision, and an afferent pupillary defect (which may be undetectable in individuals with bilateral or prior contralateral optic neuritis) are all possible symptoms of optic neuritis (6). MOG-IgG patients often experience optic disc swelling and edema, with bilateral optic neuritis more common in 45-95% (7) (**Figure 1C**). Optic neuritis can occur in all ages (7–9), and relapses are often unilateral (7, 9, 10). Despite having similar severity of neuroaxonal injury, children with MOG-IgG optic neuritis show better visual recovery than adults, as measured by various assessments including visual acuity tests, visual field tests, color vision tests, multifocal visual evoked potential (mVEP), and optical coherence tomography (OCT) (11) which provides structural information about the retina, indirectly related to predicting visual recovery.

While optic neuritis dominates the clinical features of MOGAD, involvement of other cranial nerves remains uncommon but diagnostically significant. Cobo et al. in 2019 has mentioned that the oculomotor (CN-III), trigeminal (CN-V), and vestibulocochlear (CN-VIII) neuropathies, typically presenting with diplopia, facial sensory disturbances, or auditory-vestibular symptoms respectively. These manifestations often correlate with transitional zone inflammation at central-peripheral myelin junctions (12).

Transverse myelitis is another common presentation in patients with MOG-IgG (2, 13), which can occur alone, alongside ADEM or optic neuritis (14, 15). However, some imaging characteristics such as specific patterns of spinal cord lesion location, length, and enhancement on MRI may show some trends, but these are not conclusive enough to be considered distinct differentiating features. Clinical manifestations include sensory, motor, and sphincter disturbances (14, 15), with moderate to severe acute attacks in more than 50% of patients (16). Most patients experience good motor recovery (14, 15) but may experience permanent bladder, bowel, or sexual dysfunction (13, 14).

Other less common manifestations of MOGAD include ADEM, cerebral cortical encephalitis, brainstem symptoms, and cerebellar ataxia (2, 13). ADEM is particularly prevalent in children under 10 (17, 18) while cerebral encephalitis occurs in 6-7% of MOG-IgG patients, presenting with fever, headache, and seizures (19). These clinical features can be identified as clinical indicators of MOGAD.

### 2.2 Serological biomarkers

Serological testing for MOG antibodies remains the mainstay of MOGAD diagnosis. The recently introduced MOGAD criteria depend on the seropositivity status of MOG-IgG autoantibodies (3). When testing for MOG-IgG, the serum is the recommended specimen type (18, 19) whereas the clotting factors in plasma can affect the outcome, and while CSF testing shows promise, further research is needed (18, 19). Serum testing for suspected MOGAD using cell-based assays detects secondary antibodies against IgG-Fc or IgG1 to detect MOG-IgG (20, 21). Fixed assays are an alternative, but lower sensitivity and specificity (20, 22, 23). ELISA is not recommended for MOG-IgG measurement owing to low sensitivity and specificity (20, 24). The serum testing for MOG-IgG should be done before the treatment starts (3) of MOGAD. Serum MOG-IgG reports should include both qualitative and semi-quantitative results, with low positives considered if the live assay or fixed assay results are in the low range or if the titers are at least 1:10, and less than 1:100 (3). Regular monitoring of MOG-IgG titer throughout the disease course is significant for monitoring disease activity, assessing relapse risk, and guiding treatment decisions. Persistent or rising MOG-IgG levels may indicate that the disease is still active or relapsing while declining levels could suggest remission (3). It can remain positive for years or become seronegative with or without immunotherapy. Seroconversion is rare in patients with negative test results (4, 25). If false positive, repeat testing should be done after 3 months or during relapse (3). CSF testing may support MOGAD diagnosis if clinical and MRI findings are strongly suggestive (26–28). Serum MOG-IgG titers do not only strongly predict recovery or relapse in MOG patients (3). Persistent MOG-IgG seropositivity increases the likelihood of relapse (2, 29). Dual positivity is rare, and when it occurs, AQP4-IgG titers are high, while MOG-IgG titers are low (30). Patients with NMOSD seronegative for AQP4-IgG should be tested for MOG-IgG (3) along with evidenced of clinical manifestations. MOG autoantibodies are rare in MS patients, but false positive results may occur due to high prevalence (3). Therefore, serum MOG autoantibody serves as the primary serum biomarker for the diagnosis, prognosis, and recurrence of MOGAD.

### 2.3 CSF biomarkers

When MOG-IgG seronegative patients have to support clinical and MRI characteristics, CSF testing for MOG IgG can be used in certain scenarios to support the diagnosis of MOGAD (3). Over 50% of patients with MOG-IgG and a first demyelinating event have CSF pleocytosis, which is defined by white cell counts of more than 5 cells per µL and is also more common in ADEM or transverse myelitis patients and during attacks (20, 31). Elevated CSF protein is present in 30% of patients, but does not differentiate MOG-IgG-associated demyelination from other neuroinflammatory illnesses (20, 28). Up to 20% have oligoclonal bands which does not rule out the diagnosis (20, 31).

### 2.4 Radiological biomarkers

Brain and spinal cord magnetic resonance imaging (MRI) plays a crucial role in the diagnostic workup of MOGAD. Dedicated orbital fat-saturated images of optic nerves are critical for confirming optic nerve inflammation, with key features including optic nerve head swelling, lesion extent, and perineural tissue involvement (3) (**Figure 1C**). Patients with MOG-IgG may have silent brain or optic nerve lesions, detected in 33-50% of patients with clinical transverse myelitis and MOG-IgG (14–16). Most patients with transverse myelitis associated with MOG-IgG have T2-hyperintense lesions on spinal MRI, with acute transverse myelitis often being longitudinally extensive (14–16, 18). Transverse myelitis and MOG-IgG patients experience dorsal nerve root thickening and contrast enhancement (32, 33) (**Figure 1B**). Most acute T2-hyperintense spinal cord lesions are centrally located on axial imaging, with 20-25% of lesions not involving grey matter, producing the H-sign (14, 34). Contrast enhancement is seen in 50% of patients, and cauda equina and pial enhancement have been reported. Most T2 lesions resolve or reduce in size substantially at follow-up, and spinal cord atrophy can occur in severe cases (16, 35).

Patients with MOG-IgG often have bilateral, ill-defined, and large brain lesions, often with deep grey matter involvement, unlike classic multiple sclerosis lesions (3). In MOGAD, the pons is a frequent site of involvement, typically showing large demyelinating lesions in the middle cerebellar peduncle which is a pattern distinct from MS or AQP4-IgG-seropositive NMOSD (8, 36). A small proportion of patients with brainstem lesions and MOG-IgG experience episodic but persistent nausea and vomiting, but the appearance of brainstem lesions doesn’t reliably differentiate between MOG-IgG and AQP4-IgG-seropositive NMOSD (37). Tumefactive lesions can cause life-threatening subfalcine and tentorial herniation (38) in children with MOGAD associated with ADEM (**Figure 1A**). Over 70% of cases have a complete or nearly complete clinical and radiological resolution. Still, outcomes can be poor in rare cases with leukodystrophy-like brain imaging patterns (18, 39). FLAMES, or FLAIR hyperintense lesions, in anti-MOG encephalitis with seizures, are more prominent in patients with focal or generalized seizures. In addition, relapses in ADEM patients with MOG-IgG seropositive are more common than those without seronegative (18, 40), with the time from onset to first relapse varying. Consequently, the varied radiological characteristics may serve as a biomarker for MOGAD, but further research is required. Fadda et al. (33), 2022 described that conus medullaris is most affected in MOGAD (30-40%) than in NMOSD (10-15%) and MS (5-30%). Moreover, Shahriari et al (41), 2021 has stated that conus medullaris is more common feature in MOGAD. A comparative summary of these MRI features is provided in **Table 2**.

**Table 2:** Comparative MRI Findings in MOGAD, AQP4 +NMOSD, and MS.

MRI Feature	MOGAD	AQP4+NMOSD	MS
Optic Nerve Involvement	Optic nerve head swelling, perineural enhancement, longitudinally extensive lesions	Optic neuritis (often unilateral, severe)	Short-segment optic nerve lesions, periventricular involvement
Spinal Cord Lesions	Longitudinally extensive transverse myelitis (LETM), central T2-hyperintensity, H-sign, dorsal root enhancement, conus medullaris (30-40%)	LETM common, conus medullaris (10-15%), dorsal column involvement	Short-segment lesions (<3 vertebrae), peripheral or lateral lesions, conus medullaris (5-30%)
Brain Lesions	Bilateral, large, ill-defined, deep grey matter involvement, pons/middle cerebellar peduncle lesions, ADEM-like (tumefactive in children)	Periaqueductal, hypothalamic, area postrema lesions (diencephalic pattern)	Periventricular, ovoid, Dawson’s fingers, cortical/juxtacortical lesions
Enhancement Patterns	Optic nerve, pial, cauda equina, and conus medullaris enhancement common	Patchy or ring-enhancing lesions, optic nerve enhancement	Nodular or ring-enhancing lesions (open-ring in active MS)
Brainstem Involvement	Large pontine/middle cerebellar peduncle lesions, episodic nausea/vomiting	Area postrema syndrome (intractable hiccups/nausea)	Small, focal brainstem lesions
FLAMES (FLAIR lesions)	Present in anti-MOG encephalitis with seizures	Rare	Rare
Lesion Resolution	>70% complete/near-complete resolution, rare leukodystrophy-like patterns	Partial resolution, residual atrophy common	Chronic T1 black holes, persistent lesions

## 3 Management of MOGAD

Experts recommend high-dose corticosteroids as first-line treatment for acute MOGAD attacks. A study found that intravenous methylprednisolone (IVMP) expedited visual recovery in patients with acute ON, but did not improve long-term visual outcome (42) or reduce thinning of the peri-papillary retinal nerve fiber layer (43–45). Patients with recent MOGAD attacks are at risk for relapse after discontinuing or reducing corticosteroid therapy. Relapsing optic neuritis occurs in 30-50% of patients with MOG-IgG (7, 44, 46). To reduce relapse risk, corticosteroids should be gradually tapered over 2–3 months. The recent study revealed that a daily dose of 12.5 mg of prednisone for adults (or 0.16 mg/kg/day for children) taken for at least 3 months at the initial attack of MOGAD helps delay the first relapse (47). Plasmapheresis (PLEX) and immuno-adsorption (IA) are proposed as therapeutic options for those failing IVMP treatment (48, 49). These methods have been successful in treating immune-mediated neurological diseases, including acute NMOSD attacks. However, it’s difficult to predict if a newly diagnosed patient will develop a relapsing course, as approximately 25% of MOGAD patients become seronegative and are more likely to have monophasic disease (2, 17, 50).

Cobo-Calvo et al. found that adults are at a higher risk of relapse and may experience worse clinical outcomes than children (2). Long-term immunotherapy is typically initiated after a second MOGAD attack (51). Common therapies include oral steroids, azathioprine, mycophenolate mofetil, and B cell-targeting biologics like rituximab (RTX) and IL-6 receptor antagonist like tocilizumab (51, 52). Maintenance therapy with oral corticosteroids has been associated with a reduction in relapse rate. RTX reduces relapses and EDSS scores in MOGAD (53, 54), though to a lesser extent than in NMOSD. Maintenance IVIG therapy was associated with a reduced relapse rate in adult MOGAD patients (55), and IL-6 inhibitors like tocilizumab and satralizumab (56) may be effective in MOGAD maintenance therapy. Recent studies have introduced tocilizumab as a new drug that has been used in many cases with multiple relapse cases of MOGAD in both adults and pediatrics (56–60) including life-threatening conditions. In this situation, it’s worth noting that managing MOGAD with steroids or IVIG during the initial attack may lower the risk of relapse. However, a relapse could occur as the dosage is gradually reduced. Therefore, the approach to dose adjustment could also be viewed as a potential biomarker for MOGAD.

## 4 Relapse in MOGAD

Relapse refers to a new clinical attack occurring over 30 days after a previous attack, more common in the first 6 months. They can occur within 2 months of oral corticosteroid therapy tapering or cessation (7, 13), with some patients experiencing early relapses and others experiencing ongoing relapses beyond 12 months (61). The age of the initial attack and the characteristics of the attack may influence the relapse risk but no single factor reliably predicts the progression of the disease (51). Patients with optic neuritis seem to be more likely to experience early relapses compared to those with transverse myelitis or ADEM (13, 62). A study using brain MRI scans found that clinically silent brain lesions were present in a minority of children with MOG-IgG (14% of patients, 4% of all scans). 44% of silent lesions were detected within 3 months of the first attack, and 66% within the first year (63). The study recommends not diagnosing relapsing MOGAD solely on MRI and restricting it to patients with clinically relapsing disease (3). Longitudinal MOG-IgG seropositivity is associated with relapsing MOG, but many remain seropositive and do not relapse (61, 64). Thus, the clinical features including ON, TM, and ADEM while paraclinical features with neuroimaging features, including MRI and OCT findings, may serve as biomarkers for relapse in MOGAD, distinguishing it from other neuroinflammatory disorders (65, 66). Notably, relapses in MOGAD are not reliably predicted by factors such as age, gender, race, the intensity of the initial episode, antibody titer, or treatment modalities (67).

## 5 Novel biomarkers for MOGAD

More research is required to fully understand the pathophysiology and severity of MOGAD. Identifying novel biomarkers in MOGAD is crucial for accurate diagnosis, prognosis, and treatment monitoring. Recently some of the new biomarkers have been implemented for the diagnosis of MOGAD. Lin et.al (68) found that the higher platelet-lymphocyte ratio(PLR) was linked to MOGAD relapse, and neutrophil-lymphocyte ratio(NLR) assists in distinguishing between MOGAD and MS at the onset of the disease. The temporal correlation of NLR with MOGAD activity could be used as a supportive biomarker for acute relapse (69). In addition, acute-phase MOGAD may be distinguished by the mRNA levels of higher neurofilament light chain (NFLs) in serum and CSF, decreased endothelial growth factor receptor (EGFR), and a lower EGFR/NFLs ratio in serum. The MOGAD patients’ serum and CSF have higher mRNA levels of NFLs, which aid in differentiating the ADEM-like phenotype. Furthermore, Wang et al. (70) mentioned that in pediatrics MOGAD, the serum EGFR/NFLs mRNA ratio is a good indicator of the severity of the condition. Further investigations are warranted to elucidate the pathological mechanisms underlying these associations. Moreover, Chang et al. (71) showed that sNfL and sGFAP levels are linked to disease severity in AQP4-ab-positive NMOSD and MOGAD patients, with the sGFAP/sNfL ratio indicating distinct disease pathogenesis. Ziaei et al. (72) also found high sNfL levels close to clinical or MRI events in pediatrics onset multiple sclerosis and MOGAD, supporting sNfL as a biomarker of disease activity. Concerning more, Wang et al. stated that the blood-brain barrier may become dysfunctional as a result of serum and CSF levels of IL-33, which can then cause intrathecal production of immunoglobulin in AQP4+NMOSD and MOGAD, particularly in MOGAD. It may have served as a biomarker for demyelinating disorders of the central nervous system, at least in a part (73). On the other hand, another study reveals that Th2, Th17, and CD4+ cells contribute to the pathogenesis of MOGAD in children. The recombinant human (rh)-MOG protein elicits a different response from regulatory T-cells in MOG non-relapsing (MOGNR) and MOG relapsing (MOGR), suggesting that MOGR may have lost its tolerance to the MOG autoantigen, potentially causing relapses (74). Neuroinflammatory conditions require activated microglia for demyelination, and the secreted ectodomain of soluble triggering receptor expressed on myeloid cells 2 (sTREM2) is associated with abnormal biological pathways. Cerebrospinal fluid (CSF) sTREM2 concentration is higher in neuroinflammatory and neurodegeneration diseases. Increased CSF and serum sTREM2 concentration in pediatric patients suggests microglia activation in MOGAD. CSF sTREM2 levels significantly correlated with clinical inflammatory indexes and adapted modified Rankin Scale score, indicating the potential value of sTREM2 as a severity biomarker (75).

## 6 Future directions and implications

Advances in high-throughput technologies, including genomics, proteomics, and metabolomics, hold promise for identifying novel biomarkers in MOGAD. Collaborative efforts are needed to establish large-scale longitudinal cohorts to validate candidate biomarkers and elucidate their clinical utility. Integration of multi-modal biomarker panels may enhance diagnostic accuracy and facilitate personalized therapeutic approaches in MOGAD. We can hereby find some future challenges regarding the MOGAD biomarkers in the recent published articles. Wang et al. (76) in 2024 studies using Gene Ontology, InterPro, and Kyoto Encyclopaedia Genes and Genomes identified 429 differentially expressed proteins (DEPs) in the MOG group among them 149 upregulated and 280 downregulated proteins. Functional analysis showed that the dysregulated proteins were mostly involved in cell adhesion, axon guidance, complement and coagulation cascades, and glycosphingolipid production in MOG patients. Hence, the proteomic changes seen in CSF samples from children with MOGAD that were discovered in the current study may present chances to create new biomarker candidates in the future. Additionally, Yandamuri et al. (77), in 2023 studied high-throughput assays that measured complement activity, complement-dependent cytotoxicity (CDC), antibody-dependent cellular phagocytosis (ADCP), and antibody-dependent cellular cytotoxicity (ADCC) of MOG-expressing cells. The study showed cytotoxicity is not solely dependent on MOG autoantibody quantity, engagement of effector functions is bimodal, and CDC and ADCP magnitudes increase closer to relapse. Future relapse risks can be predicted by assays that quantify CDC and ADCP. Furthermore, Liyanage et al. (78), in 2024 resulted in non-P42 MOG-IgG epitope status remained unchanged from onset throughout the disease course and was a strong predictor of a relapsing course in patients with unilateral optic neuritis and can predict a relapsing course. Patients with unilateral optic neuritis, the most frequent MOGAD phenotype, can reliably be tested for non-P42 MOG-IgG epitope at onset, regardless of age and sex. Hence, early detection and specialized management in these patients could minimize disability and improve long-term outcomes. Moreover, Ding et al. (79), in 2024 used Automated fibre quantification (AFQ) to investigate white matter damage in pediatrics MOGAD using Diffusion tensor imaging (DTI) metrics. It identified demyelination-dominated microscopic integrity damage. The study also analyzed potential neurological function alterations and their relationship with clinical disability in vulnerable fiber tracts. The microstructural injury of these fiber tracts might be the underlying cause for abnormal neurological function in MOGAD. Certain fiber tracts showed specific DTI metrics patterns, promising potential biomarkers for future research.

## 7 Conclusion

Biomarkers are promising for diagnosing and managing MOGAD patients, aiding disease progression prediction and relapse outcomes. However, more novel biomarkers are needed for future evaluation. Our study has critically summarized the possible new biomarkers for MOGAD which will help in current neuroscience.
